# Green Synthesis of Soluble Polysilsesquioxane with Phthalimide Groups

**DOI:** 10.3390/ijms25010057

**Published:** 2023-12-19

**Authors:** Artem I. Emel’yanov, Yuliya I. Bolgova, Olga M. Trofimova, Alexander S. Pozdnyakov

**Affiliations:** A.E. Favorsky Irkutsk Institute of Chemistry, Siberian Branch of the Russian Academy of Sciences, 1 Favorsky Str., 664033 Irkutsk, Russia; emelyanov@irioch.irk.ru (A.I.E.); omtrof1@irioch.irk.ru (Y.I.B.); omtrof@irioch.irk.ru (O.M.T.)

**Keywords:** polysilsesquioxane, phthalimide, hydrolytic polycondensation

## Abstract

Soluble polysilsesquioxane containing side-chain phthalimide groups (PSQ-PhI) was synthesized via a solvent- and catalyst-free hydrolytic polycondensation reaction using 2-[3-(triethoxysilyl)propyl]-1*H*-isoindole-1,3(2*H*)-dione. The composition and structure of polysilsesquioxane was confirmed via ^1^H, ^13^C, and ^29^Si NMR spectroscopy, Fourier transform infrared spectroscopy, gel permeation chromatography, thermogravimetric analysis, dynamic light scattering, X-ray diffraction analysis, and elemental analysis. The synthesized silsesquioxane showed a monomodal molecular weight distribution. The average molecular weight of polysilsesquioxane is 11,200 Da, and the polydispersity index is 1.10. ^29^Si NMR analysis showed a half-peak width w_1/2_ 3.1 ppm at δ −68.3, which corresponds to the PhI(CH_2_)_3_SiO_3/2_ unit and indicates an ordered structure in the polymer, with some defects caused by the presence of uncondensed silanol groups. PSQ-PhI showed good thermal stability (T_d5%_ decomposition at 345 °C). The polysilsesquioxane-based coating was transparent in the visible region.

## 1. Introduction

Organosilicon compounds are valuable reagents and building blocks in organic synthesis for the creation of more complex structures and occupy an important place in the field of polymers and material science [1]. An object of intensive research comprises silsesquioxanes, hybrid organosilicon materials with the general formula [RSiO_3/2_]_n_, in which each silicon atom is connected by covalent bonds to three oxygen atoms and one functional group. Silsesquioxanes are of interest due to their high thermal, chemical, and physical stability, hydrophobicity, transparency, low dielectric constant, and lack of toxicity. These properties make them promising precursors for creating new generations of materials with unique characteristics. Oligomeric and polymeric organosilsesquioxanes are used in the production of electronic devices, for the functionalization of various surfaces, the creation of additives that increase the thermal stability of materials, etc. [2,3,4,5,6,7,8,9,10,11]. For these substances, the possibility of interaction with biologically important substrates, the creation of organic–inorganic hybrid materials, biomaterials, anti-abrasive and anti-corrosion coatings, and optically active materials (including quantum dots) has been established [10,12,13,14,15,16,17,18,19]. The most important starting materials in sol–gel chemistry are alkoxysilanes, which can be polymerized via hydrolysis and condensation to form various polysiloxane materials [10,13,14]. The nature of the substituents framing the silicon atom largely determines the properties and structure of organosilicon monomers and polymers. In this regard, the synthesis of structurally controlled alkoxysilanes is of paramount importance.

Recently, materials based on silsesquioxanes have found a wide range of applications in biomedicine [20]. Polyhedral oligomeric silsesquioxanes (POSSs) are non-toxic and have good biocompatibility [21,22]. In addition, it was found that POSS cages, as nanoscale building blocks, can be incorporated into other polymers with improved mechanical and viscoelastic properties [23,24,25]. The incorporation of POSSs into poly(carbonate-urea) urethane improves biological stability, which is one of the most important factors when selecting polymers for medical use, particularly for the manufacture of vascular grafts and shunts [23,24,25,26,27]. The incorporation of POSSs into poly(methyl methacrylate) not only largely overcomes the weaknesses of conventional commercial acrylic dental composites, but also improves their biocompatibility [21,28]. Olivero and et al. [29] reported the synthesis of a luminescent POSS, containing, in its structure, a fluorescein derivative and a carboxyl group, suitable for fixing various organic compounds (dyes, markers, drugs, contrast agents). Moreover, POSS-based systems, due to their small size, can be easily transferred into the cell compartment, making these samples promising carriers of biological molecules and suitable for biomedical applications [30]. POSSs are also widely used for gene and targeted drug delivery, the detection of biological macromolecules (DNA and peptides), as contrast agents in MRI or optical imaging, and for the development of biomedical devices and tissue engineering scaffolds [31,32,33].

Heterocyclic compounds play an important role as the basis for many bioactive molecules. Most nitrogen-containing heterocycles are widely used in medicine, industry, and agriculture. Among imides, the most important are cyclic imides—derivatives of dicarboxylic acids. Thus, phthalimide, succinimide, glutarimide, saccharin, etc., are widely used in the organic synthesis of heterocyclic compounds, amines, amino acids, and peptides. Phthalimide derivatives have therapeutic and antitumor activity, are included in antibiotics, analgesics, and cholinesterase inhibitors used in Alzheimer’s disease, and are used as immunomodulators [34,35,36,37,38].

Thus, the functionalization of organosilicon compounds via introducing a cyclic imide paves the way for the creation of functionalized organosilicon polymer systems, giving them new properties and expanding the possibilities of their use.

Previously, the synthesis of oligomeric phthalimide-functionalized silsesquioxanes obtained via the reaction of nucleophilic substitution of the chlorine atom in octakis(3-chloropropyl)octasilsesquioxane with a phthalimide group in DMF was reported [39]. Mono- and trifunctional siloxane oligomers were prepared via the reaction of the N-3-(trimethoxysilylpropyl)phthalimide with hexamethyldisiloxane or divinyltetramethyldisiloxane in the presence of methanol and HCl [40]. Kaneko et al. reported the synthesis of polysilsesquioxane containing phthalimide groups via the two-step modification of ammonium-group-containing polysilsesquioxane [41]. The reaction was carried out through reacting it with phthalic anhydride in the presence of DMSO, triethylamine, and an aqueous solution of hydrochloric acid to obtain PSQ containing phthalamic acid side-chain groups. In the next step, the precursor was refluxed in DMF at 160 °C to obtain polysilsesquioxane containing phthalimide side-chain groups.

Currently, much attention is being paid to carrying out chemical reactions that take into account the principles of “green chemistry” formulated by Anastas and Warner [42]. These principles assume the absence of the use of organic solvents or the use of water as a medium, avoiding the preparation of intermediate products, achieving the maximum conversion of the starting compounds during the reaction into the resulting product, and reducing the number of process stages, including, among other things, improving the simplicity of chemical transformations with high efficiency.

In this work, we present a facile and environmentally friendly method for the synthesis of soluble polysilsesquioxane with side-chain phthalimide groups. PSQ-PhI was prepared via a widely used hydrolytic polycondensation reaction of trifunctional alkoxysilane in the absence of any solvents or catalysts. The composition, structure, and properties of PSQ-PhI are discussed using techniques such as FTIR and NMR spectroscopy, gel permeation chromatography (GPC), X-ray diffraction analysis (XRD), thermogravimetric analysis (TGA), and dynamic light scattering (DLS). The synthesized polysilsesquioxane has high thermal stability and the ability to form transparent polymer films.

## 2. Results

**Scheme 1:**
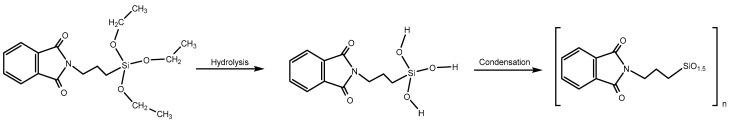
Synthesis route of polysilsesquioxane (PSQ-PhI).

2-[3-(Silsesquioxanyl)propyl]-1*H*-isoindole-1,3(2*H*)-dione (PSQ-PhI) was synthesized via a simple and efficient method, using the hydrolytic polycondensation reaction of the trifunctional silane monomer 2-[3-(triethoxysilyl)propyl]-1*H*-isoindole-1,3(2*H*)-dione containing hydrolytically active ethoxy groups, in accordance with the principles of green chemistry (absence of organic solvent, reaction at atmospheric pressure, maximum conversion of the initial compound during the reaction into the resulting product) (Scheme 1).

**Figure 1:**
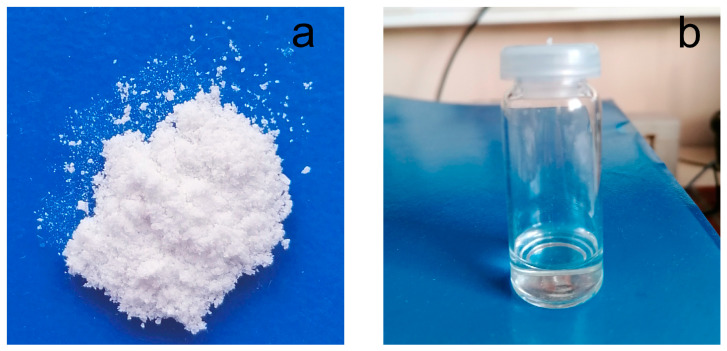
Images of the PSQ-PhI (**a**) and 2-[3-(triethoxysilyl)propyl]-1*H*-isoindole-1,3(2*H*)-dione (**b**).

The reaction was carried out in water for 8 h at 70 °C. During hydrolysis, the ethoxy groups of trifunctional alkoxysilane are hydrolyzed to form silanol. Further, the reactive trisilanol undergoes a homo- and heterofunctional condensation reaction, leading to polysilsesquioxane, the main chain of which consists of a Si–O–Si bond skeleton, in which the silicon atom is bound to the phthalimide moiety by a propylene bridge. As a result of the reaction, the target PSQ-PhI was isolated as a white powder with a yield of ca. 97% (Figure 1). The resulting polysilsesquioxane containing side-chain phthalimide groups is soluble at room temperature in various organic solvents (chloroform, alcohols, acetone, DMF, DMSO). To assess the solubility of silsesquioxane, 0.1 g of PSQ-PhI was added to 1 mL of the corresponding solvent. Within 1–2 min, the solution became homogeneous. The solubility of PSQ-PhI in water was 0.2 mg per 1 mL.

**Figure 2:**
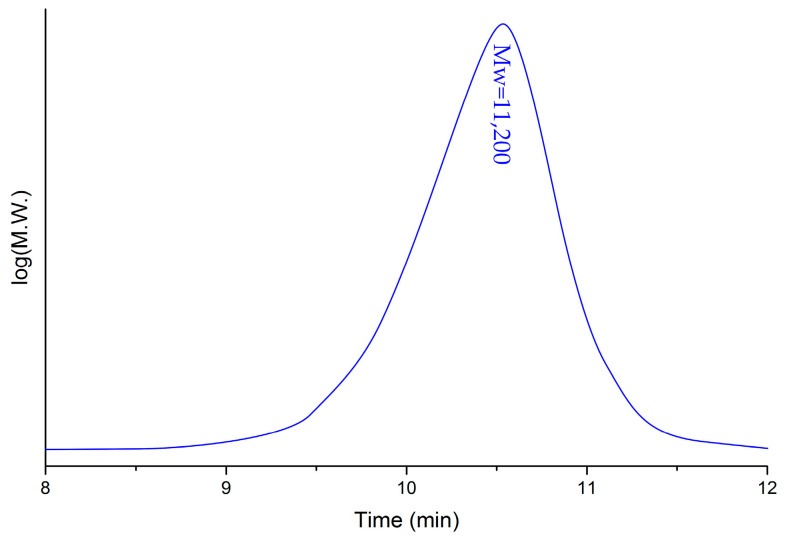
Gel permeation chromatography curve of PSQ-PhI.

The molecular weight and molecular weight distribution of polysilsesquioxane were characterized using gel permeation chromatography. The weight average molecular weight of PSQ-PhI is 11,200 Da, with a polydispersity index of 1.10. As can be seen from the GPC curve (Figure 2), the synthesized polysilsesquioxane have a monomodal molecular weight distribution.

It is worth noting that the molecular weights of PSQ-PhI obtained via GPC are indicative values based on the use of narrow dispersed polystyrene standards, taking into account the difference between their hydrodynamic volumes. However, the results suggest that PSQ-PhI has a narrow polydispersity. Based on this, it can be assumed that the resulting silsesquioxane has a relatively uniform size distribution and probably consists of fully or incompletely condensed cage-ladder structures [43,44]. The previously described method for obtaining polysilsesquioxane containing phthalimide side-chain groups by modifying ammonium-group containing polysilsesquioxane led to the formation of silsesquioxane with a polydispersity index Mw/Mn of 3.19 [41]. Our proposed approach provided the synthesis of PSQ-PhI with a very narrow polydispersity (1.10), which is essential for many practical applications, especially in the field of biomedical applications.

Molecular weight and polydispersity are important characteristics of polymers that affect their properties. High-molecular-weight polymers generally have better mechanical properties, while lower-molecular-weight polymers have better solubility [45]. Molecular mass characteristics significantly impact the processes of elimination of polymers from the body, the ability to pass through the blood–brain barrier, prolonged drug release, toxicity, etc. The narrow fractional composition of polymers can ensure the stability of polymer properties. Therefore, the molecular-weight characteristics of polymers are important for each specific biomedical application [46].

**Figure 3:**
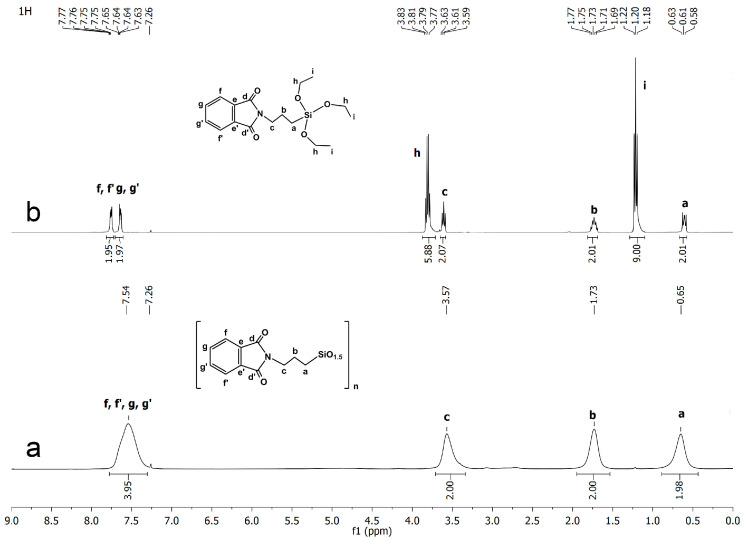
^1^H NMR spectrum of 2-[3-(silsesquioxanyl)propyl]-1*H*-isoindole-1,3(2*H*)-dione (**a**) and 2-[3-(triethoxysilyl)propyl]-1*H*-isoindole-1,3(2*H*)-dione (**b**).

The molecular structure of the isolated silsesquioxane was characterized via ^1^H, ^13^C, and ^29^Si NMR spectroscopy. As shown in the ^1^H NMR spectrum (Figure 3), the resonance signals in the ranges of 3.83–3.77 ppm and 1.22–1.18 ppm, corresponding to the methylene and methyl protons of the ethoxy group, disappeared, indicating the complete hydrolysis of the monomer. At the same time, broader signals of the phthalimide moiety methylene protons and alkyl chain protons, compared with the sharp peaks in the ^1^H NMR spectrum of the initial monomer, indicate that the triethoxysilane has condensed into a relatively high-molecular-weight silsesquioxane, as confirmed using GPC data.

**Figure 4:**
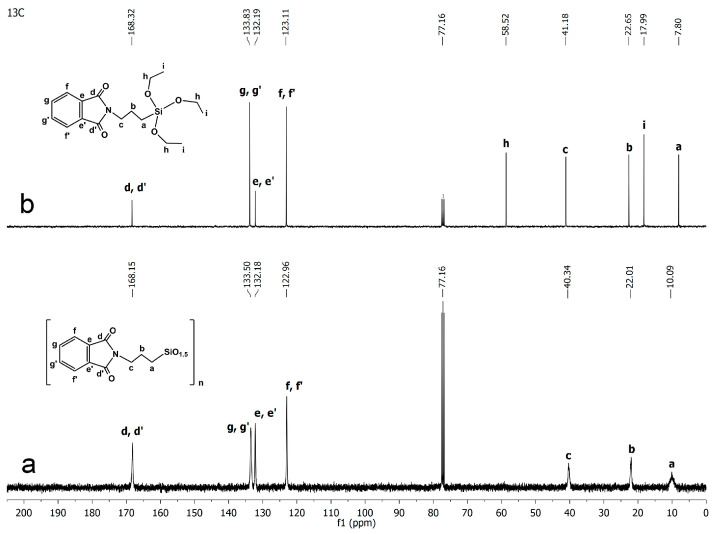
^13^C NMR spectrum of 2-[3-(silsesquioxanyl)propyl]-1*H*-isoindole-1,3(2*H*)-dione (**a**) and 2-[3-(triethoxysilyl)propyl]-1*H*-isoindole-1,3(2*H*)-dione (**b**).

The ^13^C NMR spectrum (Figure 4) also confirms the polymeric nature of the synthesized polysilsesquioxane. The spectrum shows broader intense peaks of phthalimide fragments and -CH_2_-CH_2_-CH_2_-Si. There are no signals at 58.52 ppm or 17.77 ppm attributed to the monomer alkoxysilyl groups.

**Figure 5:**
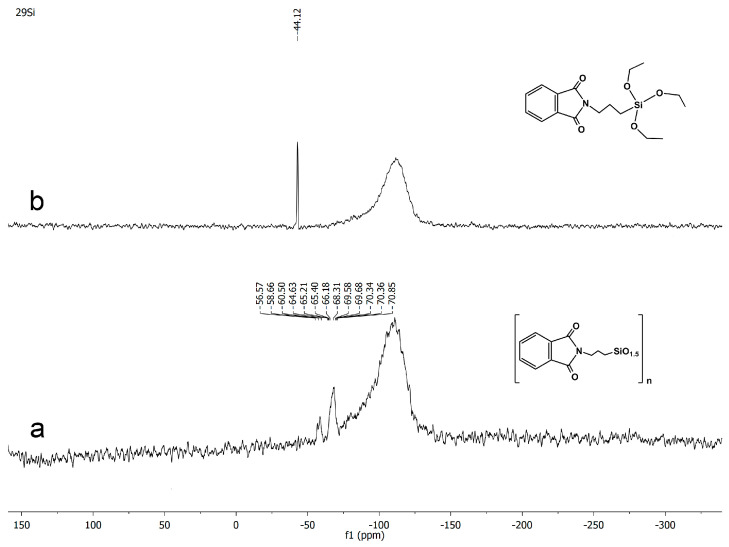
^29^Si NMR spectrum of 2-[3-(silsesquioxanyl)propyl]-1*H*-isoindole-1,3(2*H*)-dione (**a**) and 2-[3-(triethoxysilyl)propyl]-1*H*-isoindole-1,3(2*H*)-dione (**b**).

According to the literature data, there are four groups of silicon atoms with different degrees of condensation [47,48,49]. The degree of condensation for silicon atoms with three hydroxyl or three alkoxy substituents without Si–O–Si siloxane bonds is denoted as T^0^. Silicon atoms with one siloxane bond and two hydroxyl groups are denoted as T^1^, and silicon atoms with two siloxane bonds and one hydroxyl group are denoted as T^2^. The designation T^3^ corresponds to fully condensed silicon atoms with three Si–O–Si siloxane bonds. From the ^29^Si NMR spectrum of PSQ-PhI (Figure 5), it is clear that there are two clearly defined peaks for the fully condensed silicon atom T^3^ (PhI(CH_2_)_3_SiO_3/2_) and the partially condensed silicon atom T^2^ (PhI(CH_2_)_3_(OH)SiO_2/2_) with centers at −68.3 and −58.7 ppm, respectively. Peaks T^0^ and T^1^ are not observed. The broad T^3^ peak for silsesquioxane suggests a variety of structures such as ladder, cage, and defective branched structures [50]. The T^3^ peak from −64.6 to −70.9 ppm is associated with the dominant microstructures, whereas the T^2^ peak from −56.6 to −60.5 is associated with the ends of the ladders.

The integrating of the T^2^ and T^3^ signals allows us to calculate the ratio of T units and, consequently, the degree of condensation, in accordance with the following equation [47]:

DC [%] = (3·T^3^ [%] + 2·T^2^ [%])/3 (1)

The calculated degree of condensation of the PSQ-PhI was 90%. According to the literature [49], the percentage of residual OH groups is calculated using the proportion of cross-linked silicon atoms (DC) based on the assumption that the non-bridging oxygen atoms belong to either ethoxy groups or silanol groups:

OH [%] = 100% − DC [%] − OEt [%](2)

For the synthesized silsesquioxane, the amount of residual OH groups was 10%.

The value of the chemical shift of the silicon atoms of the T^3^ units of the polymer skeleton in combination with the width of the resonance peak at half height (w_1/2_) can serve as a fairly reliable identifier of the ladder structure of polysilsesquioxane; the narrower w_1/2_, the higher the structure regularity. The half-peak width (w_1/2_) 3.1 ppm indicates the presence of structural defects in the backbone ladder chain of the synthesized PSQ-PhI.

**Figure 6:**
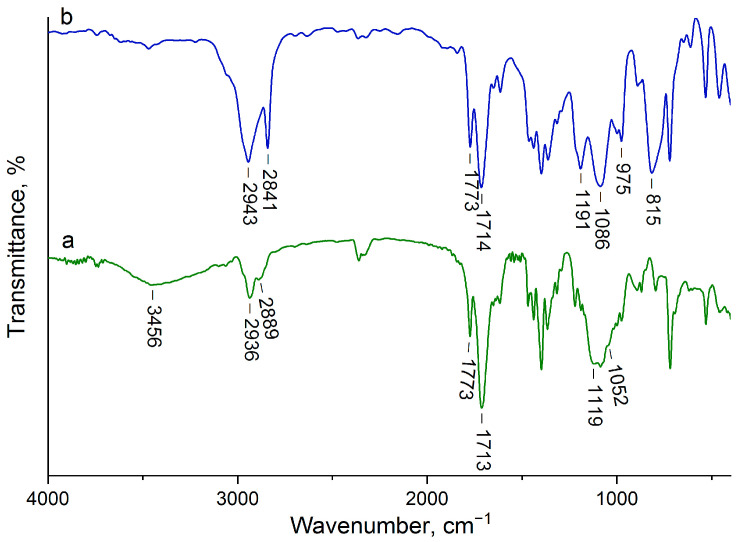
FTIR spectrum of 2-[3-(silsesquioxanyl)propyl]-1*H*-isoindole-1,3(2*H*)-dione (**a**) and 2-[3-(triethoxysilyl)propyl]-1*H*-isoindole-1,3(2*H*)-dione (**b**).

Figure 6 shows the FTIR spectra of polysilsesquioxane and its triethoxysilane precursor 2-[3-(triethoxysilyl)propyl]-1*H*-isoindole-1,3(2*H*)-dione.

Compared with the monomer, the ethoxysilyl group peaks at 1191 and 815 cm^−1^ completely disappeared, confirming the completion of the hydrolysis reaction [51]. A broad absorption band from 3600 to 3000 cm^−1^, with a maximum at approximately 3456 cm^−1^, corresponds to the stretching vibration of associated hydroxyl groups, which indicates the existence of incompletely condensed silanol bonds. The phthalimide group remains unaffected by the hydrolysis and condensation reaction. This is confirmed by the presence absorption peaks at 1773 cm^−1^ (ν_s_ C=O) and 1713 cm^−1^ (ν_as_ C=O) and 1468–1438 cm^−1^ and 1397–1366 cm^−1^ (C=C, C–C bond deformation vibration phthalimide cycle). The absorption bands at 2936 cm^−1^ and 2889 cm^−1^ are attributed to the C–H stretching vibration of the alkyl group. The change in the character of the absorption bands in the 1200–1000 cm^−1^ region confirms the occurrence of the condensation process. The strong broad band in this region, resulting from the stretching vibrations of the Si–O–Si bond, demonstrates the presence of different structural species of silsesquioxane [52,53]. In particular, the 1119 cm^−1^ band is assigned to the symmetrical cage structure. Broader multiple siloxane peaks are seen at 1000–1100 cm^−1^, indicating the possibility of the presence of ladder and random structures. However, according to the literature data [54], the IR spectra indicate that the synthesized PSQ-PhI has an incompletely condensed cage-ladder structure.

**Figure 7:**
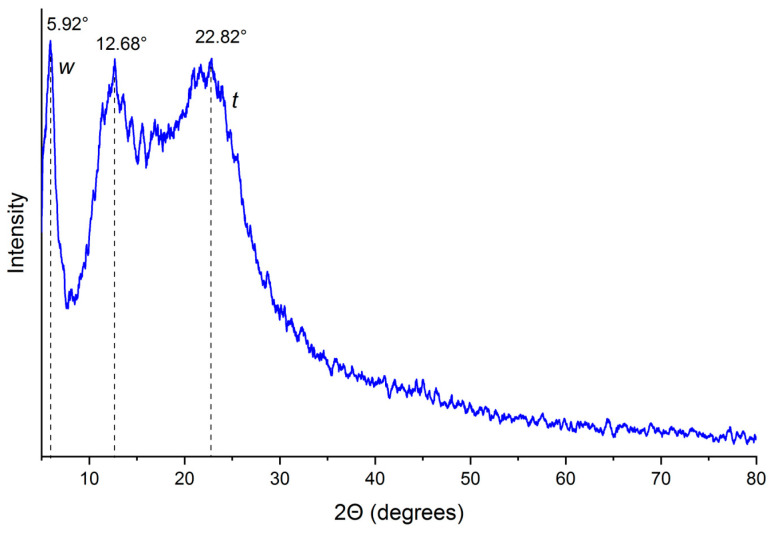
X-ray diffraction pattern of polysilsesquioxane.

The structure of polysilsesquioxane with side-chain phthalimide groups in the solid state was investigated using powder X-ray diffraction analysis. Figure 7 displays the XRD pattern of the powder sample of the synthesized silsesquioxane for 2θ from 5° to 80°. There are three maxima in the XRD pattern of PSQ-PhI at 2θ = 5.92°, 12.68°, and 22.82°, corresponding to the periodic distances 14.92 Å, 6.98 Å, and 3.89 Å. The shape of the diffraction curve is characteristic of the amorphous nature of the polymer. However, a sharp diffraction peak (w) at 2θ = 5.92°, assigned, according to the literature data [55,56,57], to the intramolecular chain-to-chain distance (or the width of each double chain) in a double-chain ladder molecule of silsesquioxane, indicates a fairly regular arrangement of PSQ-PhI macromolecules. The broad peak (t), covering a wide range of 2θ diffraction angles from 9° to 30°, indicates the average thickness of the Si–O–Si structure of the ladder macromolecules. PSQ-PhI has two types of distances in the siloxane backbone (6.98 Å and 3.89 Å), which is likely due to its cage-ladder structure [54].

**Figure 8:**
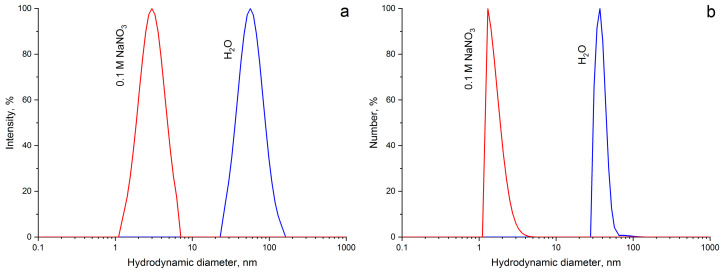
Hydrodynamic diameter distribution by intensity (**a**) and by number (**b**) for PSQ-PhI at 0.1 mg/mL.

The dynamic light scattering (DLS) method was used to measure the effective dynamic diameters of polymer coils (D_h_) of 2-[3-(silsesquioxanyl)propyl]-1*H*-isoindole-1,3(2*H*)-dione in water and aqueous salt solution. The dynamic light scattering spectra of PSQ-PhI are shown in Figure 8. According to the obtained curves of the dependence of the signal intensity on the hydrodynamic diameter in water and aqueous salt solution, the synthesized polysilsesquioxane forms a colloidal system with a monomodal particle size distribution. At the same time, the sizes of scattering particles corresponding to the hydrodynamic diameter of silsesquioxane differ in water and aqueous salt solution.

**Figure 9:**
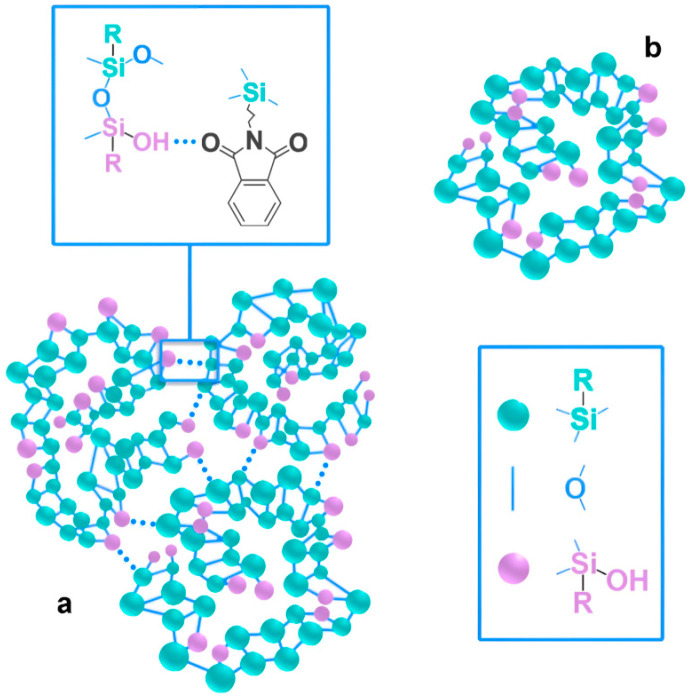
2-[3-(Silsesquioxanyl)propyl]-1*H*-isoindole-1,3(2*H*)-dione: (**a**) in water (associated state); (**b**) in aqueous salt solution (unassociated state).

In water, polysilsesquioxane is in an associated state, with a particle diameter from 27 to 68 nm. The data obtained indicate that silsesquioxane macromolecules in water are in a bound state, forming supramolecular structures due to the intermacromolecular interaction of individual macromolecules. The formation of such associated structures occurs due to the formation of hydrogen bonds with the participation of carbonyl oxygen atoms of the imide fragment and hydroxyl groups at the silicon atom that belong to different macromolecular chains (Figure 9). According to ^29^Si NMR spectroscopy, the content of Si–OH groups is up to 10% of the total amount of silicon. With an increase in the ionic strength of the solution in a water–salt medium, macromolecular associates disintegrate into individual polymer chains, the hydrodynamic diameter of which is in the range from 1 to 5 nm.

**Figure 10:**
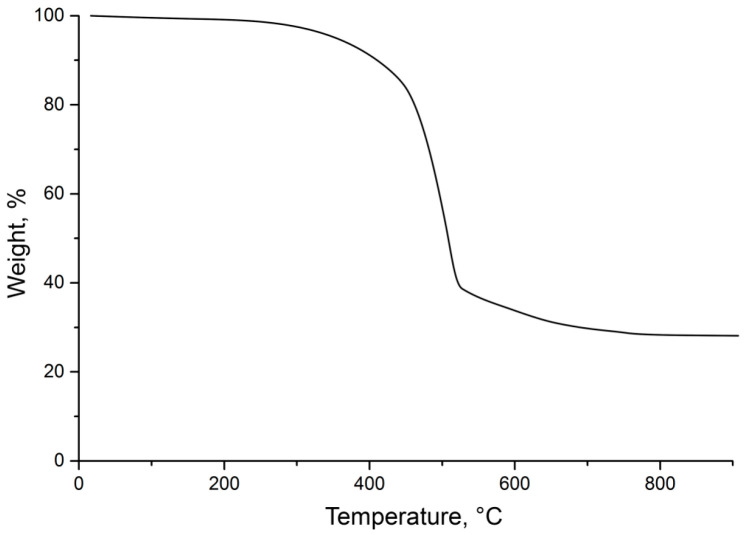
Thermogravimetric curve of PSQ-PhI in air.

The thermal stability of the synthesized polysilsesquioxane was studied using thermogravimetric analysis in an oxidizing atmosphere. The TGA curve of PSQ-PhI is shown in Figure 10.

Polysilsesquioxane showed high thermal stability. The temperatures for 5% mass loss and 10% mass loss were 345 °C and 408 °C, respectively. These values are higher than those of polysilsesquioxane containing phthalimide side-chain groups (318 and 343 °C) previously obtained by Kaneko et al. [41]. The slight mass loss is probably caused by the condensation of residual hydroxyl groups accompanied by the release of water. The thermal oxidative destruction of silsesquioxane in air is characterized by two stages of weight loss. The first stage, corresponding to a significant weight loss (52%) occurs at temperatures from 408 °C to 520 °C, which is likely due to the decomposition of the phthalimidopropyl organic substituent. The second stage of weight loss (8–10%) at temperatures from 520 °C to 750 °C corresponds to further thermal oxidative destruction of the unstable char layer formed by the phthalimide ring and the alkyl chain. At temperatures above 750 °C, no further change in weight was observed. The resulting stable solid residue is 28% and probably corresponds to the residue of silica and carbon formed during the complete decomposition of silsesquioxane.

**Figure 11:**
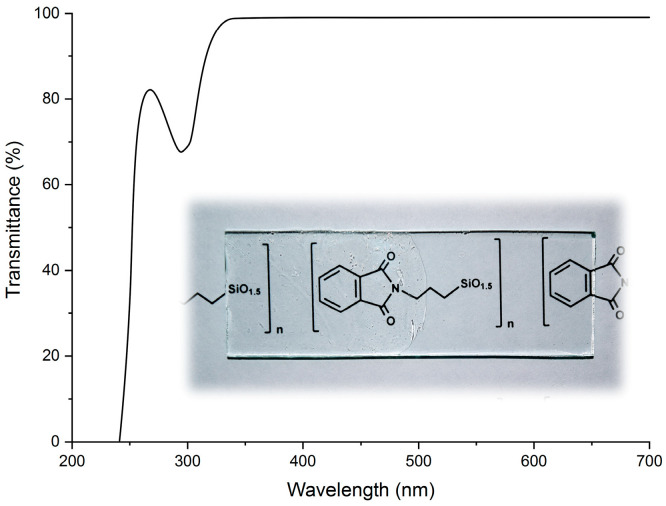
UV–Vis spectrum of PSQ-PhI film cast on quartz glass and photograph (inset) of a polymer-coated glass substrate.

In order to characterize the optical properties of polysilsesquioxane, a film based on it was drop-cast from a 1 wt. % DMSO solution on a glass flat substrate and left for slow evaporation during the day under ambient conditions. After drying at 80 °C for 6 h, a transparent and colorless film was formed. The transmittance of the PSQ-PhI-based film was measured in the 200–700 nm wavelength range using UV–Vis spectroscopy (Figure 11). The PSQ-PhI film on a glass substrate showed high transparency in the visible region of the spectrum. The absorption in the ultraviolet region, with a maximum at 287 nm, belongs to the carbonyl chromophore groups of the phthalimide fragments of the synthesized silsesquioxane.

**Figure 12:**
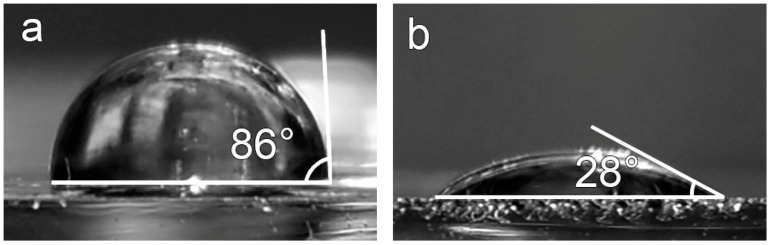
Photographs of water contact angles: (**a**) on the surface of a PSQ-PhI film loaded on a silicate glass, (**b**) on the surface of silicate glass without treatment.

The hydrophobic–hydrophilic properties of the PSQ-PhI-film-coated surface were assessed using contact angle measurements. The contact angle θ when placing a 5 μL water drop on a polysilsesquioxane film cast from a DMSO solution was 86° (Figure 12a), which is significantly higher than the water contact angle with the hydrophilic surface of silicate glass without any treatment (*θ* = 28°) (Figure 12b). These data indicate sufficiently high water-repellent properties in the resulting PSQ-PhI polymer film.

## 3. Materials and Methods

### 3.1. Materials

Phthalimide potassium salt (98%) was used as purchased from Sigma-Aldrich (Munich, Germany). The starting (3-chloropropyl)triethoxysilane was purchased from Sigma-Aldrich (Munich, Germany). Deionized water (resistivity ≥ 17.5 MΩ∙cm, Vodoley-M water purifier, RU) was used for the silsesquioxane synthesis.

### 3.2. Synthesis of 2-[3-(triethoxysilyl)propyl]-1H-isoindole-1,3(2H)-dione monomer

(3-Chloropropyl)(triethoxy)silane (23.77 g, 98.4 mmol) was added dropwise to a solution of the 1*H*-isoindole-1,3(2*H*)-dione potassium salt (18.22 g, 98.4 mmol) in DMF (50 mL) in the presence of dibenzo-24-crown-8 ether (0.035 g, 0.08 mmol). The reaction mixture was stirred magnetically at 80 °C for 2.5 h. The formed precipitate was filtered. The filtrate was distilled under a vacuum. Triethoxysilane precursor was obtained with a yield of 95% (32.86 g), T_b_ = 195 °C (1.00 torr). Anal. Calc. (%) for C_17_H_25_NO_5_Si: C 58.09, H 7.17, N 3.99, Si 7.99; Found (%): C 54.72, H 7.15, N 4.18, Si 8.07. FTIR (ν, cm^−1^): 2943 (CH_2_), 2841 (C–H in Me), 1773, 1714 (C=O), 1650, 1614 (C=C, 1*H*-isoindole-1,3(2*H*)-dione), 1463, 1438 (C=C, C–C), 1397, 1362 (C=C, C–C), 1315 (C–N), 1191, 1086, 975 (SiOC), 815 (SiOC), 721 (=C–H). ^1^H NMR, δ, ppm: 7.77–7.75 (m, 2H, H-f,f’), 7.65–7.63 (m, 2H, H-g,g’), 3.83–3.77 (q, ^3^*J* = 7.1 Hz, 6H, OCH_2_), 3.63–3.59 (t, 2H, ^3^*J* = 7.6 Hz, NCH_2_), 1.77–1.69 (m, 2H, CH_2_–C*H*_2_–CH_2_), 1.22–1.18 (t, ^3^*J* = 7.1 Hz, 9H, CH_3_), 0.63–0.58 (m, 2H, SiCH_2_). ^13^C NMR, δ, ppm: 168.32 (C=O), 133.83 (C-g, C-g’), 132.19 (C-e, C-e’), 123.11 (C-f, C-f’), 58.52 (OCH_2_), 41.18 (NCH_2_), 22.65 (CH_2_–*C*H_2_–CH_2_), 17.99 (CH_3_), 7.80 (SiCH_2_). ^29^Si NMR, δ, ppm: −44.1.

### 3.3. Synthesis of 2-[3-(silsesquioxanyl)propyl]-1H-isoindole-1,3(2H)-dione (PSQ-PhI)

The monomer 2-[3-(triethoxysilyl)propyl]-1*H*-isoindole-1,3(2*H*)-dione was polymerized using deionized water (pH 7) without the addition of a catalyst and without the use of a solvent. Deionized water (3 mL) was added dropwise to the monomer (0.309 g, 1.0 mmol) with stirring. Next, the reaction mixture was heated to 70 °C and stirred vigorously at this temperature for 8 h. After the mixture was cooled to room temperature, the aqueous alcohol layer was decanted. The remaining transparent viscous substance was dried in a vacuum at room temperature for 24 h to give the target silsesquioxane containing phthalimide side-chain groups, as a white powder with m.p. 85–88 °C (0.201 g, yield, ca. 97% based on the ideal chemical formula of one unit of this product [C_11_H_10_NO_3.5_Si, FW = 240.294]). Anal. Calc. (%) for C_11_H_10_NO_3.5_Si: C 54.98, H 4.19, N 5.83, Si 11.69; Found (%): C 55.05, H 4.16, N 5.28, Si 12.17. FTIR (ν, cm^−1^): 3456 (O–H), 2936, 2889 (CH_2_), 1773, 1713 (C=O), 1468–1438, 1397–1366 (C–C, C=C, 1*H*-isoindole-1,3(2*H*)-dione moiety), 1200–1000 (Si–O–Si). ^1^H NMR, δ, ppm: 7.54–7.26 (br, 4H, H-f,f’,g,g’), 3.57 (br, 2H, NCH_2_), 1.73 (br, 2H, CH_2_–C*H*_2_–CH_2_), 0.65 (br, 2H, SiCH_2_). ^13^C NMR, δ, ppm: 168.15 (C=O), 133.50 (C-g, C-g’), 132.18 (C-e, C-e’), 122.96 (C-f, C-f’), 40.34 (NCH_2_), 22.01 (CH_2_–*C*H_2_–CH_2_), 10.09 (SiCH_2_). ^29^Si NMR, δ, ppm: −56.6 to −60.5 (T^2^) and −64.6 to −70.9 (T^3^).

### 3.4. Characterization

FTIR spectra were recorded on a Spectrum Two FTIR spectrometer (PerkinElmer, Shelton, CT, USA) with an attenuated total internal reflection sensor at room temperature in the wavenumber range of 400–4000 cm^−1^ with a resolution of 4 cm^−1^ and 16 scans.

The ^1^H, ^13^C, and ^29^Si NMR spectra were obtained using a Bruker DPX-400 spectrometer (Bruker, Bremen, Germany) at 400.13, 100.62, and 79.50 MHz, respectively. The NMR spectra were measured at 297 K in CDCl_3_. Standard 5 mm glass NMR tubes were used. For the ^1^H and ^13^C NMR spectra, chemical shifts are indicated relative to the residual protons of the solvent CDCl_3_ (7.26 and 77.16 ppm, respectively). ^29^Si chemical shifts are assigned relative to the external standard TMS (0 ppm). The average molecular weight and molecular weight distribution were determined via gel permeation chromatography using the Shimadzu LC-20 Prominence system (Shimadzu Corporation, Kyoto, Japan) fitted with a Shimadzu RID-20A differential refractive index detector and Agilent PolyPore 7.5 × 300 mm (PL1113-6500) column at 50 °C. *N*,*N*-Dimethylformamide was used as an eluent. The prepared sample was weighed and dissolved in DMF at room temperature for 24 h with stirring. The concentration of the solution was about 10 mg/mL. The flow rate was 1 mL/min. Calibration was carried out using a series of polystyrene standards from Polystyrene High EasiVials (PL2010-0201), consisting of 12 samples with molecular weights from 162 to 6,570,000 g/mol. Deionized water solution and water–salt solution (NaNO_3_ 0.1 M) with 0.1 mg/mL silsesquioxane concentration were used to determine the hydrodynamic particle diameter (D_h_) of the investigated sample via dynamic light scattering using a ZetaPALS Potential Analyzer equipped with BI-MAS module (Brookhaven Instruments Corporation, Holtsville, NY, USA). The measurements were carried out in a thermostatted cuvette with an operating temperature of 25 °C and an angle of detection of scattered light equal to 90°, and at a wavelength of 659 nm. The average particle diameter was obtained by averaging the values obtained from three series of measurements of 10 scans each.

The hydrodynamic particle diameter was calculated using the Stokes–Einstein relation, according to which the diffusion velocity is inversely proportional to the particle size.

D = k_B_T/3πηD_h_ (3)

where k_B_ is the Boltzmann constant (1.380 × 10^−23^ kg·m^2^·s^−2^·K^−1^), T (K) is an absolute temperature, η (kg·m^−1^·s^−1^) is the viscosity of medium, D_h_ (m) is the hydrodynamic particle diameter, and D (m^2^·s^−1^) is the diffusion coefficient.

Thermogravimetric analysis was determined using a TGA *i* 1000 Thermogravimetric Analyzer from Instrument Specialists Inc. (Twin Lakes, WI, USA) under air atmosphere at 5 °C/min from 20 to 900 °C. The weight of the samples was 7 mg.

X-ray powder diffraction data were obtained using a D8 ADVANCE Bruker diffractometer (Bruker Corporation, Boston, MA, USA) with a CuKα radiation source (λ = 1.5406 Å) and equipped with a scintillation detector.

The scans were performed in the range of diffraction angles 2θ from 5° to 80° with a step size of 0.02°. The experiments were carried out at room temperature in the Bragg–Brentano geometry with a flat sample. Each exposure time was set to 2 s; the operating current and voltage were 40 mA and 40 kV, respectively. The interplanar spacings w and t were calculated using the Bragg equation [58]:

nλ = 2dsinθ(4)

where n is a positive integer called the reflection order, λ is the X-ray wavelength, d is the spacing of the planes, and θ is the Bragg angle (the glancing angle at which the X-rays are reflected).

The optical transmittance of the PSQ-PhI-based film was determined in the 200–700 nm wavelength range using a Lambda 35 UV/Vis spectrophotometer (PerkinElmer, Shelton, CT, USA).

Elemental analysis was performed with a Thermo Scientific Flash 2000 CHNS-Analyzer (Thermo Fisher Scientific, Cambridge, UK). Gravimetric determination of silicon content was carried out via the literature method [59].

## 4. Conclusions

Thus, the work presents an easy and environmentally friendly approach, taking into account the principles of “green chemistry” (no organic solvents, catalysts, close-to-quantitative yield of the target product, one-pot synthesis method), to the synthesis of soluble polysilsesquioxane with side-chain phthalimide groups. Hydrolytic polycondensation provided polysilsesquioxane with an average molecular weight of 11 kDa, with a narrow polydispersity index of 1.10. Using IR, NMR, and X-ray diffraction methods, it was established that the synthesized PSQ-PhI has an incompletely condensed cage-ladder structure. Using the dynamic light scattering method, it was established that silsesquioxane macromolecules in water are in an associated state, forming supramolecular structures due to the intermacromolecular interaction of individual macromolecules. PSQ-PhI has high thermal stability (345 °C) and the ability to form transparent polymer films. The presence of residual silanol groups in the synthesized incompletely condensed polysilsesquioxane provides various opportunities for its further modification and wide practical application.

The proposed approach for obtaining PSQ-PhI, based on the accessible methodology of hydrolytic polycondensation, in terms of the set of synthesis parameters (medium, temperature, time) and characteristics (polydispersity, thermal stability) differs favorably from currently known methods for obtaining post-modified PHQ.

## Data Availability

Data are contained within the article.
